# Sphingosine-1-Phosphate as Lung and Cardiac Vasculature Protecting Agent in SARS-CoV-2 Infection

**DOI:** 10.3390/ijms241713088

**Published:** 2023-08-23

**Authors:** Manale Karam, Christian Auclair

**Affiliations:** AC BioTech, Villejuif Biopark, Cancer Campus, 1 mail du Professeur Georges Mathé, 94800 Villejuif, France; manale.karam@gmail.com

**Keywords:** sphingosine-1-phosphate, SARS-CoV-2, COVID-19, lung vasculature

## Abstract

Severe acute respiratory syndrome coronavirus 2 (SARS-CoV-2) may cause severe respiratory illness with high mortality. SARS-CoV-2 infection results in a massive inflammatory cell infiltration into the infected lungs accompanied by excessive pro-inflammatory cytokine production. The lung histology of dead patients shows that some areas are severely emphysematous, with enormously dilated blood vessels and micro-thromboses. The inappropriate inflammatory response damaging the pulmonary interstitial arteriolar walls suggests that the respiratory distress may come in a large part from lung vasculature injuries. It has been recently observed that low plasmatic sphingosine-1-phosphate (S1P) is a marker of a worse prognosis of clinical outcome in severe coronavirus disease (COVID) patients. S1P is an angiogenic molecule displaying anti-inflammatory and anti-apoptotic properties, that promote intercellular interactions between endothelial cells and pericytes resulting in the stabilization of arteries and capillaries. In this context, it can be hypothesized that the benefit of a normal S1P level is due to its protective effect on lung vasculature functionality. This paper provides evidence supporting this concept, opening the way for the design of a pharmacological approach involving the use of an S1P lyase inhibitor to increase the S1P level that in turn will rescue the lung vasculature functionality.

## 1. Introduction—Abnormal Vasculature in SARS-CoV-2-Associated Lung Pathogenesis

Coronavirus disease 2019 (COVID-19) is a respiratory disease caused by a coronavirus, i.e., severe acute respiratory syndrome coronavirus 2 (SARS-CoV-2), a positive single-stranded RNA virus that leads to substantial morbidity and mortality. SARS-CoV-2 infection causes symptoms of a severe respiratory illness similar to SARS-CoV and MERS-CoV (Middle East respiratory syndrome-related coronavirus). SARS-CoV-2 infects its host by first binding respiratory epithelium in the upper airways [1]. At this time, the host may remain asymptomatic or present non-specific flu-like symptoms, i.e., fatigue, fever, and rhinorrhea. The virus then migrates to the lower airways, where it binds to a receptor on the cell membrane (i.e., angiotensin-converting enzyme 2 (ACE2)) allowing viral uptake within the cell, thereby inducing systemic infection [1]. Clinical evidence indicates that the virus-infected cell-mediated immune response associated with the uncontrolled production of inflammatory cytokines, known as a cytokine storm or cytokine release syndrome, is responsible for most severe illnesses that patients can experience when infected with SARS-CoV-2, often resulting in alveolar injury that reduces airway capacity and in multi-organ failure [2]. By better understanding the mechanisms that drive the intensity of the cytokine storm, we can develop treatment strategies to prevent the severe conditions and fatal outcome of this disease.

Substantial evidence indicates that lung pathogenesis associated with SARS-CoV-2 infection is characterized by abnormal vasculature involving the dysfunction of endothelial cells (ECs) and mural cells. In fact, COVID-19 results in lung imaging abnormalities including emphysematous large areas, with enormously dilated blood vessels and micro-thromboses, as observed by lung histology of patients that died after SARS-CoV-2 infection [3,4,5,6]. In many cases, diffuse alveolar lesions are evident, with peeling of the pneumocytes, formation of hyaline membranes, and fibrotic exudates. Moreover, symptomatic COVID-19 patients present strong inflammatory responses characterized by inflammatory cell infiltration including monocytes, macrophages, and neutrophils. Damage to the pulmonary interstitial arteriolar walls indicates that inflammatory response plays an important role in the development of the disease. These deleterious immune responses have been related to a “cytokine storm” [7,8], characterized by an increased plasma concentration of many pro-inflammatory cytokines and chemokines such as interleukin (IL)-1β, interleukin-1 receptor antagonist (IL-1Rα), IL-2, IL-6, IL-7, IL-8, IL-9, IL-17, and tumor necrosis factor α (TNF-α) [7,8,9]. This hyper-inflammatory state has a direct deleterious effect on the vascular system with resulting EC dysfunction. In the presence of circulating inflammatory mediators such as IL-1 and IL-6, ECs undergo an evolution into an activated condition that generally participates in host defenses [10]. Activated ECs induce localized inflammation by activating pro-inflammatory gene expression and recruitment of inflammatory cells, and vascular leak by increasing endothelial permeability [11]. Furthermore, EC injury is worsened by toll-like receptor activation caused by viral RNA recognition, which results in an increase in reactive oxidative species production [12,13].

The presence of dilated pulmonary capillaries in SARS-infected areas [14] suggests an alteration of pericytes, which are contractile cells linked to vascular smooth muscle cells. The pericytes envelop the capillaries, forming close contacts with the adjacent ECs [15]. Pericytes are characterized by the presence of markers, including platelet-derived growth factor (PDGF) a and b receptors and sphingosine-1-phosphate (S1P) receptor 1. Pericytes also express neural/glial antigen 2 (NG2) proteoglycan, CD146, collagen I(α)1, smooth muscle α actin (α-SMA), vimentin, and desmin [16].

Pericytes express ACE2, the target of the spike protein (S protein) responsible for virus internalization in host cells [17]. Although in the lung, unlike other organs, the majority of pericytes appeared to be ACE2 negative, particularly in the alveolar region, ACE2-positive pericytes were found near the larger bronchi and abundant in the capillaries of the trachea [18]. Additionally, pericytes expressing ACE2 are present in large quantities in the heart. The dysfunction of cardiac pericytes could take part in the evolution of cardiovascular abnormalities linked to viral infection, such as fibro-calcifying cardiovascular remodeling, arterial hypertension, myocardial edema, and coronary no-reflow phenomenon. Of note, exposure of pericytes to recombinant protein S alone causes signaling and functional alterations, including (i) a reduction in the capacity to promote the formation of an EC network in three-dimensional Matrigel; (ii) the release of pro-inflammatory molecules; and (iii) the synthesis of pro-apoptotic factors, causing EC death [19]. These observations suggest that pericytes are cellular targets of SARS-CoV-2, which induces the alteration of their functions, thus participating in vascular abnormalities in different organs.

In the heart and lungs of COVID-19 patients, vascular coverage by pericytes is strongly decreased compared to healthy individuals without decreased capillary density, indicating that SARS-CoV-2 may negatively impact the microvasculature by preferentially targeting pericytes [20,21].

## 2. The Control of S1P Metabolism as a Strategy of Choice to Efficiently Treat COVID-19 Patients by Protecting Lung Vasculature

The suggested pivotal role of ECs and pericytes in COVID-19 aggravation suggests that vascular normalization strategies throughout the deleterious immune response could be beneficial. Several clinical trials have explored the effect of targeting molecules involved in vasculature abnormalities in acute lung injury in COVID-19 patients. Among these molecules, angiopoietin 2 (Ang2) was targeted (clinical trial number NCT04342897), with the reasoning that circulating Ang2 levels correlate with increased pneumonic edema and mortality in patients with acute respiratory distress syndrome including COVID-19 [22,23,24,25]. Other clinical trials (NCT04275414, NCT04305106 and NCT04344782) have explored bevacizumab, a monoclonal antibody that binds and inhibits the major vascular permeability inducer, i.e., vascular endothelial growth factor (VEGF), that increases pulmonary edema during infection and that is also implicated in the induction of coagulation signaling pathways in severe COVID-19 [26]. Yet, the results of only one (NCT04275414) of these trials have been published [27]. The administration of bevacizumab in combination with the standard of care in patients with severe COVID-19 pneumonia demonstrated clinical efficacy by ameliorating oxygenation and shortening the duration of oxygen support. Despite the presumably adverse effects of VEGF inhibitors [28], the results of this clinical study strongly support the hypothesis that targeting vasculature abnormalities in acute lung injury is greatly beneficial for patients with severe COVID-19.

**Figure 1:**
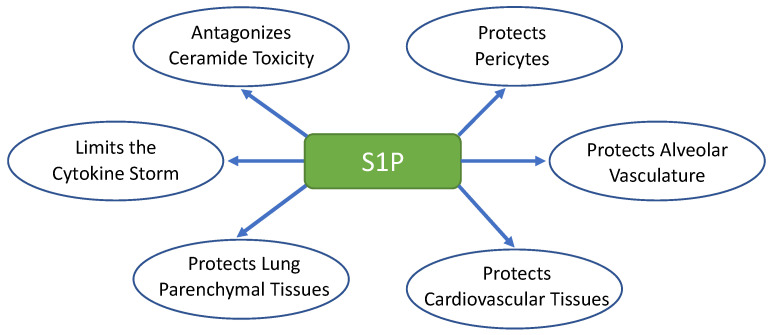
Sphingosine-1-phosphate (S1P), a key regulator of immune cell trafficking, inflammation, and vascular integrity.

Normalization of the vascular wall by metabolic intercessions through a pharmacological approach could be designed as a new therapeutic approach. For instance, ECs treated with drugs that target prime metabolic enzymes of the glycolytic pathway acquire improved vascular integrity and reduced ischemia and leakiness, thereby adopting a ‘normalized’ phenotype [29]. Here, given the biological properties of S1P that plays an important role in vascular normalization and regulation of inflammation (Figure 1), we hypothesize that targeting S1P’s metabolism may represent an appropriate strategy to fight against inflammation and normalize the lung vasculature. This proposal is further supported by a publication indicating that a low plasmatic S1P level is a marker for a worse prognosis in severe COVID-19 [30].

### 2.1. S1P General Properties

S1P is one of the most abundant biologically active lysophospholipids in the circulation [31,32]. At the cellular level, it is present in all mammalian cells, where it acts as a second messenger in signal transduction pathways to modulate cellular processes such as cell differentiation, apoptosis, proliferation, migration, and adhesion [31,33]. S1P is also an agonist of five G-protein-coupled receptors (*S1P1-S1P5*) [34]. S1P receptors are coupled differentially via G(i), G(q), G(12/13), and Rho to numerous effectors, which include the phospholipases C and D, adenylate cyclase, extracellular-signal-regulated kinase, p38 mitogen-activated protein kinase, c-Jun N-terminal kinase, and nonreceptor tyrosine kinases. Autocrine and paracrine interactions between S1P and its receptors can regulate a wide range of physiological processes including angiogenesis, resistance to apoptosis, and inhibition of lymphocyte egress from primary and secondary lymphoid tissues, resulting in the depletion of recirculating lymphocytes from the peripheral blood [31]. 

### 2.2. S1P and S1P Lyase in Immune Cell Trafficking

S1P-dependent trafficking of hematopoietic cells, including lymphocytes, dendritic cells, macrophages, and neutrophils, is a crucial process whereby they can migrate between secondary lymphoid organs and the peripheral organs that may be subject to inflammation, infection, or injury. This process depends on the cell type and activation state and on the expression of S1P receptors, their ligation, and downstream signaling events. The most well-described process is that of T cell egress from secondary lymphoid organs [35]. Trafficking of mature CD4 and CD8 single-positive T cells from the thymus is essential for the development of adaptive immune responses and the limitation of auto-reactive T cells from entering the circulation [35]. These cells use S1P chemotactic gradients to migrate out of primary and secondary lymphoid organs with low S1P levels to the plasma and/or lymph with high S1P levels and on towards the periphery [35,36]. Mature thymocytes express the transcription factor Krüppel-like factor 2, which upregulates its target gene S1PR1. The activation of S1PR1 by S1P enables the mature T cells to migrate in response to minute quantities of S1P in the nanomolar range, thereby exiting the thymus and entering the circulation [35,36,37,38]. This upregulation of S1PR1 is associated with a reduced expression of the chemokine receptor CCR7, that is involved in the homing of T cells to secondary lymphoid organs [39]. The binding of S1P to the cognate S1PR1 and its subsequent internalization on activated T cells initiate downstream signaling pathways for actin-dependent cytoskeletal rearrangements, integrin clustering, and downregulation of CD69. This contributes to gaining an effector phenotype and cellular movement [35,36]. The inhibitors of S1P lyase, the enzyme that irreversibly degrades S1P, THI (2-acetyl-4-tetrahydroxybutylimidazole), and FTY720 (an S1PR1,3-5 functional antagonist) display transient lymphopenia, likely due to impaired S1P gradient generation or the inability to respond to S1P, respectively [40,41].

### 2.3. Protective Role of S1P in Lung Pathologies

A protective role for S1P in lung pathologies such as lipopolysaccharides (LPS)-induced lung injury (sepsis), pulmonary fibrosis, and bronchopulmonary dysplasia has been well described in the literature [42]. Murine and canine models indicate that administration of S1P, or its analogs, reduces vascular leakage and pulmonary edema in sepsis-induced lung injury. Furthermore, LPS administration intratracheally modulates the expression of sphingosine kinases (SphKs) and S1P lyase that catalyze S1P formation and degradation, respectively [42]. S1P lyase inhibition or SphK1 activation in the lung tissue abrogates inflammatory cytokine production and attenuates disease severity [42]. These effects may be due to a modulation in the phosphorylation of key molecules of the NFkB and MAPK pathways, as was shown in LPS-stimulated human lung microvascular endothelial cells [43].

### 2.4. S1P Antagonizes Ceramide-Induced Lung Toxicity

All sphingolipid species and their respective catalytic enzymes have been reported in the lung, consisting mainly (70%) of ceramides displaying 16- or 24-carbon fatty acid chains [44]. Ceramides play a crucial role in the regulation of vascular permeability and the evolution of acute and chronic lung injuries, particularly during platelet-activating factor (PAF)-induced lung edema [45]. Ceramides enhance vascular permeability and reduce alveolar integrity by inhibiting the surface-tension-lowering capacities of natural surfactant [46]. Upon stimulation by TNF-α, alveolar ceramides increase and alter the surfactant and epithelial permeability [46]. Furthermore, ceramides are pro-apoptotic second messengers which can stimulate apoptosis and oxidative stress in ECs and, consequently, intensify lung injury [47].

A recent study by Khodadoust (2021) has concluded a causal relationship between the concentration of ceramide in the plasma and symptoms of respiratory distress in COVID-19 patients. Lipidomic analysis of plasma samples from 52 individuals infected with COVID-19 showed a 400-fold increase in the concentration of lipids belonging to the ceramide class in the total plasma of infected patients [48]. 

S1P can antagonize ceramide-induced lung toxicity by enhancing EC survival and stabilizing the endothelial barrier (as described below) but also by stabilizing the lung epithelial barrier [49,50]. 

### 2.5. S1P Displays Angiogenic Properties and Stabilizes Microvessels

S1P signaling via specific S1P receptors, mainly S1PR1 and to a lesser extent S1PR2 and S1PR3, is a potent effector of vascular cell adhesion and motility, which are important for blood vessel formation and maturation [51,52,53,54]. S1P can induce the proliferation and recruitment of pericytes to the capillaries by stimulating the expression of PDGF-β chain [51]. PDGF-β can bind and activate its receptor PDGFR on pericyte precursors inducing their proliferation, maturation, migration, and recruitment to the capillaries [55]. In addition to recruiting pericytes to the capillaries, S1P can promote intercellular interactions between ECs and between ECs and smooth muscle cells (particularly pericytes) that are necessary for the stabilization of blood vessels, thereby reducing their permeability and leakage [52,53,56,57]. 

S1P is also closely linked to PDGF signaling. PDGF induces the expression of SphK1 protein via Egr-1 and subsequent S1P production [58,59]. PDGF and S1P act via PDGFR-β-S1P1 receptor complexes to promote mitogenic signaling and pulmonary artery smooth muscle cell proliferation [60].

Another key aspect of the effect of S1P in angiogenesis relates to its ability to counteract VEGF angiogenic function. ECs in the vascular sprouts secrete VEGF, which suppresses PDGFR-β signaling by inducing VEGFR2/PDGFR-β complexes. This pathway abolishes the coverage of endothelial sprouts by pericytes, resulting in vascular instability and regression. S1P promotes interactions between VE-cadherin and VEGFR2, which suppresses VEGF signaling (VEGF-induced angiogenic vascular sprouting) and restores PDGFR-β signaling (inducing stabilization of new vascular connections) [61,62]. Thus, S1P counteracts VEGF function either indirectly, by inducing pericyte proliferation and recruitment to the blood vessels and enhancing EC–EC and EC–pericyte cell adhesion, or directly, through VEGFR2 sequestration. VEGFR2 sequestration inhibits VEGF angiogenic signaling on the one hand and releases PDGFR-β on the other hand, thereby inhibiting sprouting angiogenesis and enhancing blood vessel junctional stability.

### 2.6. S1P’s Effects on Blood Vessel Maturation and Vascular Stabilization by Vasculature Protection in the Context of Acute Inflammation

Endothelial barrier dysfunction causes pronounced increases in vascular permeability, which is a major characteristic of inflammation and atherosclerosis [63,64,65,66]. S1P signaling plays a crucial role in the maintenance of vascular barrier integrity. For instance, mutant mice which selectively lack S1P in plasma through inducible sphingosine kinases 1 and 2 double-deletion system, exhibit basal vascular leak and reduced survival following anaphylactic reaction to platelet-activating factor or histamine [67]. This increased vascular leak correlated with expanded gaps between ECs in the venules and could be reversed by restoring plasma S1P levels through transfusion with wild-type erythrocytes or through treatment with an S1PR1 agonist. These results strongly suggest that plasma S1P maintains basal vascular integrity under homeostatic conditions via S1PR1 and prevents fatal responses to vascular-leak-inducing mediators [68]. 

Accordingly, it has been further observed that the administration of S1PR1 antagonists such as fingolimod (or FTY720) causes S1PR1 degradation and leads to vascular integrity loss and leakage in the lungs and skin [69]. Lee et al. (1999) showed that S1P induces reorganization of the actin cytoskeleton and localization of VE-cadherin and α-/β-/γ-catenins to the sites of intercellular contact together with adherens junction assembly in human umbilical vein endothelial cells [53]. S1P promotes human and bovine pulmonary artery and lung microvascular EC barrier integrity through Rac- and Rho-dependent actin cytoskeletal rearrangement, inducing endothelial cortical actin assembly with the recruitment of the actin filament regulatory protein, cofilin [70]. Thus, in the lung, S1P maintains vascular integrity by regulating important processes of rearrangements of the actin cytoskeleton and assembly of adherens junctions in the ECs [71].

In the circulation, two chaperone proteins bind S1P (~65% high-density lipoproteins-apolipoprotein M (HDL-ApoM) and ~35% albumin). In the time course of inflammation, when human primary ECs were challenged, with TNF-α acting as an inflammatory stimulus, the presence of recombinant S1P bound to its major carrier apolipoprotein M (ApoM-bound S1P) decreased the expression of adhesion molecules (VCAM-1 and E-selectin) on the cell surface. The stimulation of S1PR1 was enough and necessary to induce anti-inflammation [72]. Furthermore, ApoM-bound S1P activated the rearrangement of S1P-related genes’ expression to withstand TNF-α. At the functional level, ApoM-bound S1P inhibited the adhesion of monocytes to the endothelium and preserved endothelial barrier integrity during inflammation. Thus, ApoM-bound S1P is a critical protective factor in the endothelium, regulating adhesion molecule expression, leukocyte–endothelial adhesion, and endothelial barrier integrity.

In agreement with the ApoM-S1P proposed effects, it has been observed that, in human sepsis, the disease severity was correlated with decreased S1P levels, a profile mimicking that of plasma ApoM. Interestingly, in the plasma from severe sepsis subjects, an almost complete loss of S1P and ApoM was demonstrated in the high-density lipoprotein fractions [73]. In patients with untreated familial hypercholesterolemia, the occurrence of higher S1P and ApoM levels compared to healthy individuals, and their correlations with HDL subfractions and inflammatory markers, imply their possible role in endothelial protection [74]. In fact, HDL-associated S1P is bound specifically to ApoM which, by delivering S1P to the S1P1 receptor on endothelial cells, causally contributes to the anti-atherogenic and vasculoprotective functions of HDL [75,76,77]. In critically ill COVID-19 patients, total S1P serum levels are strongly reduced, which is significantly related to the decrease in erythrocytes, the major cellular source of circulating S1P, as well as of the two key S1P transporters ApoM and albumin. A carrier-changing shift from albumin to HDL-ApoM was also observed in critically ill patients with SARS-CoV-2 infection [78] and in patients with surgical trauma and sepsis [79]. The switch of S1P transporters from albumin to HDL with the associated drop in albumin levels could function as an adaptive response mechanism to preserve normal homeostasis. Compared to albumin-S1P, HDL-S1P reduces lymphopoiesis, neuroinflammation, and vascular inflammation, and prolongs endothelial barrier enhancement via a more efficient activation, trafficking, and signaling of S1PR1 [80]. Significantly, HDL-S1P was shown to promote sustained endothelial barrier stabilization, which is disrupted in severe COVID-19 patients. Therefore, the favored use of HDL as the predominant S1P carrier appears to be a compensatory response to adjust for decreased albumin and albumin-S1P levels so as to preserve EC barrier function and avoid an excessive inflammatory response. Furthermore, the serum levels of S1P, erythrocytes, ApoM, and albumin were also inversely correlated with admission to an intensive care unit and patient mortality [30]. Thus, low circulating S1P is a negative biomarker of the severity/mortality of COVID-19 patients and restoring abnormal S1P levels may have the potential to be a therapeutic target in patients with COVID-19.

## 3. Pharmacological Perspectives

### 3.1. Pharmacological Targeting of the S1P/S1PR Axis

It is obvious that the S1P/S1PRs axis is involved in the modulation of many physiological processes, highlighting their potential functions in pathophysiology and diseases. The significant and fast developing field of targeting S1PRs in inflammation and multiple sclerosis has been considerably reviewed [68,81,82,83,84,85]. The development of fingolimod, an inverse agonist of S1PR1 for the treatment of multiple sclerosis was a successful application of the S1P signaling pharmacology [86,87]. In contrast to multiple sclerosis, the efficacy of fingolimod in COVID-19 is still controversial. Although it was suggested that fingolimod may be beneficial as an adjuvant therapy to prevent or mitigate the severity of COVID-19 disease by preventing the rise in cytokine production at earlier stages of SARS-CoV-2 infection and by enhancing endothelial barrier function at low doses [88,89,90], at high doses and/or at prolonged exposure, fingolimod increased lung vasculature leakage [69]. The beneficial effect of fingolimod was suggested based on the observation that COVID-19 patients who were already being treated with FTY720 for their multiple sclerosis did not develop any symptoms or complications [89]. However, in vitro and in vivo preclinical studies showed that in contrast to S1P, the administration of the S1PR1 antagonist fingolimod leads to S1PR1 degradation and subsequently induces the loss of capillary integrity and vascular leakage in the lungs and skin [69,91,92,93]. More recently, a clinical trial aiming to evaluate the effect of fingolimod in COVID-19 patients showed that fingolimod does not affect patient intubation or mortality nor does it enhance the outcomes of patients with moderate COVID-19 [94]. However, fingolimod could significantly reduce the re-admission rate after hospitalization with COVID-19 [94]. Thus, the therapeutic relevance of FTY720 in COVID-19 is complex, probably due to its antagonistic effect on S1PR1 leading to the degradation of this receptor and inhibition of S1PR1-mediated vascular protection, while it can still enhance the EC barrier via a novel S1PR1-independent mechanism [88,92]. Thus, additional studies should be performed to assess the effect of fingolimod in COVID-19. 

An increased S1P level in lymph nodes, achieved through S1P lyase inhibition, has been attempted as well for the treatment of rheumatoid arthritis. For both indications, the rationale is based on the inhibition of lymphocytes’ egress from the lymph nodes, limiting the lymphocyte-mediated inflammation. The critical point is to identify pathological situations for which a plasmatic/tissue S1P increase would be beneficial for patients. The observation indicating that a low plasmatic S1P level is observed in severe SARS-CoV-2 infection highlights the interest of pharmacological action leading to an S1P level increase. Moreover, it has been reported that low expression of S1PR1 correlates with a poor prognosis in breast cancer patients [95], whereas a recent paper by Karam et al. 2022 indicates that a high plasma S1P level was associated with a significant increase in the overall survival of patients having a metastatic pancreatic adenocarcinoma treated by gemcitabine [54]. These observations, taken together with the anti-inflammatory and tissue- and microvessel-protective roles of S1P, make it obvious that a pharmacological strategy aiming to increase the S1P level may have therapeutic applications. In this order of idea, as previously mentioned, the increase in the concentration of S1P in the organs and the circulating blood can be obtained by the inhibition of S1P lyase, which is the major enzyme of degradation of S1P.

S1P lyase catalyzes the irreversible cleavage of S1P at the C2-3 carbon bond, giving rise to a long-chain aldehyde and phosphoethanolamine. S1P lyase activity is found in all mammalian tissues except for platelets and erythrocytes. Although each of these enzyme classes is present in most mammalian cells, their relative abundance varies by tissue and cell type. In addition, cells have different capacities to discharge S1P stores into the extracellular environment. The over-expression of S1P lyase induces apoptosis in response to apoptotic stimuli, results in diminished intracellular S1P levels, and increases stress-induced ceramide production. 

It must be noted that a few S1P lyase small molecular inhibitors have been already characterized. The vitamin B6 antagonist, 4′-deoxypyridoxine (4-DP) [96], and the food colorant, 2-acetyl-4tetrahydroxybutylimidazole (THI) [97] have been widely used to block S1P lyase activity, in vitro and in vivo, respectively, but have no therapeutic application. On the other hand, a THI derivative identified as LX2931 [98], also named LX3305 as a crystal form, was found to be a specific S1P lyase inhibitor and has been developed for the treatment of rheumatoid polyarthritis by Lexicon Pharmaceuticals, Inc., US (IND granted in 2007). The beneficial effect of an increased S1P level on vascular functionality could be observed in an experimental mouse model of pancreatic cancer that is characterized by extreme hypoxia linked to a strong vascular anomaly, particularly a very high vascular permeability [54,99]. In fact, in pancreatic cancer mouse models, oral treatment with LX2931 increases the level of circulating S1P [54] and results in a very significant reduction in tumor hypoxia [54] and vascular leakage (Supplementary File S1), suggesting restored vascular functionality. This beneficial effect of S1P on the tumor vasculature normalization and hypoxic status leads to reduced tumor invasion, enables drug delivery into the tumor, and improves anti-tumor immunity and response to radiotherapy [54]. However, S1P could also contribute to tumor progression by enhancing cancer cell proliferation and survival [54,100]. Thus, although the S1P lyase inhibitor LX3305 was found to be very well tolerated with no serious side effects in five phase 1 randomized double-blind placebo-controlled studies conducted by Lexicon Pharmaceuticals (USA) in healthy volunteers (four studies) (Protocol # LX3305.1-101/102/103-NRM, LX3305.1-105-NRM, and LX3305.1-201-RA-EudraCT2009-012705-19) and in rheumatoid arthritis patients (one study) (Protocol # LX3305.1-104-DDI), its potential use in COVID-19 patients with cancer should be controlled.

There is an accumulation of evidence indicating a relationship between vascular leakage and pericyte density/functionality. For example, experimental depletion of pericytes in post-natal retinal blood vessels resulted in abnormal and leaky vasculature. Recent work has identified a role for pericytes in amplifying NS1-induced microvascular hyperpermeability in severe dengue [101]. Tumor vessel leakage is well documented in experimental tumor models and human cancers. In addition to affecting the internal environment of the tumors, mainly its inflammation state, blood vessel leakiness also governs the tumor invasion capability and in turn the occurrence of metastasis. Pericytes with α-smooth muscle actin are expressed on most tumor vessels, but they are abnormally associated with endothelial cells. Excess VEGF, which can induce the generation of highly abnormal vessels in experimental models, is a potential primary contributor to vascular abnormalities. Accordingly, S1P signaling, in connection with PDGF, antagonizes this deleterious effect and restores pericyte functionality. This explanation is strongly supported by the effect of the S1P lyase inhibitor LX2931 on the restoration of vascular functionality in the pancreatic tumor model [54] (Supplementary File S1), opening a way for a pharmacological strategy aiming to restore vasculature functionality in different pathological circumstances including viral infection such as SARS-CoV-2 infection. Moreover, it should be pointed out that this strategy could be applied in other situations, such as radiotherapy-induced vasculature toxicity.

### 3.2. Experimental Animal Models for Lung Vasculature and Pathogenesis in SARS-CoV-2 Pharmacology Research

The pharmacological validation of the efficacy of S1P lyase inhibitor in restoring lung vasculature functionality and reducing pulmonary inflammation and pathology in SARS-CoV-2 infection requires appropriate animal models that recreate the clinical and pathological characteristics of COVID-19 in humans. Several animal models have been used and developed to study SARS-CoV-2 viral pathogenesis, transmission, therapeutic agents, and vaccines [102,103]. Those include mouse models (that could be sensitized to SARS-CoV-2 infection by introducing human ACE2 (hACE2) expression through transgenic, knock-in, viral–vector transduction, or virus adaptation strategies), the golden Syrian hamster model, the ferret model, the nonhuman primate (NHP) models, and the cat model. In contrast to the mouse models, the other cited models are naturally susceptible to SARS-CoV-2 infection owing to the high degree of similarity between hamster/ferret/NHP/cat ACE2 and human ACE2 [102,103]. Each model presents various advantages and disadvantages for their use in SARS-CoV-2 research that should be considered depending on the intended goal of the study [102,103]. Those benefits and limitations are related to (1) differences in the expression level of ACE2 and cellular tropism of the virus, (2) presence or absence of pulmonary and/or extrapulmonary infection symptoms and/or other clinical symptoms (weight loss, fever), (3) variations in host immune response (inflammatory markers, seroconversion, immune cell activation), (4) fatality of the infection, (5) different viral load, clearance and/or transmission, (6) demographic differences (different susceptibilities to the SARS-CoV-2 infection based on gender and age of the animal), (7) the cost, availability, ease of manipulation, behavior, maintenance, and housing, and (8) ethical and legal regulations in scientific research of the animal models [102,103]. Among these COVID-19 animal models, those that can be useful in studying the effect of S1P lyase inhibition on lung vasculature functionality, pathology, and inflammation are those that were found to reproduce the histopathological lung disease in a dose-dependent manner as was seen in humans, i.e., the hACE2-expressing mouse models (K18-hACE2 transgenic mouse model [104] and adenovirus hACE2 mouse model [105]), the Syrian hamster model [106], and the NHP models (cynomolgus macaques [107,108], rhesus macaques [109], African green monkeys [110], and baboons [111]). The other models, mouse ACE2 promoter with human ACE2 coding sequence [112], endogenous mouse ACE2 promoter model [113], and the ferret model [114], would not be useful in the study of lung clinical symptoms as they showed no or mild symptoms after infection with SARS-CoV-2 compared to the hACE2 transgenic mouse, Syrian hamster, and NHP models. Despite the usefulness of the latter models in studying the lung vasculature and pathogenesis in SARS-CoV-2 infections, they present some limitations. Although NHPs present the advantage of having a close physiological relation to the human immune response during infections, their use in animal research in more difficult. In fact, this animal model has a special legal status that necessitates strong justification for its use, and is very costly as NHPs must be kept in social groups, need larger enclosure spaces, have a more varied and costly diet, and need to be accustomed to housing and transportation to reduce stress levels, which can jeopardize scientific data. The cost and ethical and practical considerations make the hACE2-expressing mouse models and the Syrian hamster model more affordable and practical models to use; however, they also present several limitations. For example, the Syrian hamster model shows fast clearance of the SARS-CoV-2 virus and absence of death by COVID-19, limiting their usefulness in studying the fatality of the disease [106]. On the other hand, the hACE2-expressing mouse models represent a risk of ectopic hACE2 expression, which changes the cellular tropism of the virus, and the expression of hACE2 at non-physiological levels. Furthermore, both mouse and hamster models might not respond to treatments similarly to humans and, therefore, data obtained in these models might not be reproducible in humans, which is a general limitation of the experimental preclinical models. Balancing cost, practicality, safety, mimicking human COVID-19, and robustness of the animal model, the K18-hACE2 transgenic mouse model and the Syrian hamster models currently represent the suitable and affordable preclinical experimental models that reproduce the histopathological lung disease and vascular events induced by SARS-CoV-2 infection as seen in humans.

## 4. Conclusions

COVID-19 is a vascular disease where lung vasculature injuries are responsible for a large part of the SARS-CoV-2-induced lung damage and respiratory distress. The abnormal vasculature characterizing lung pathogenesis associated with SARS-CoV-2 infection involves the dysfunction of ECs and pericytes leading to vascular leakage, damaged pulmonary interstitial arteriolar walls, and promoted localized inflammation by inducing pro-inflammatory gene expression and recruitment of inflammatory cells. In the heart and lungs of human patients with COVID-19, vascular coverage by pericytes is strongly decreased, further supporting the effect of SARS-CoV-2 on these mural cells and the lung and heart microvasculature. Given the central role of ECs and pericytes in COVID-19 escalation, we propose a novel therapeutic approach involving the increase of a bioactive sphingolipid with vascular normalizing, anti-inflammatory, and anti-apoptotic properties, i.e., S1P. A significant piece of molecular and clinical evidence supports the role of S1P in protecting vascular functionality and controlling inflammation. Furthermore, decreased plasma levels of S1P is a marker of a worse prognosis of clinical outcome in severe COVID-19 patients. Increased plasma S1P could be achieved by inhibiting S1P lyase, the enzyme responsible for S1P’s irreversible catabolic pathway, and may rescue lung vasculature functionality and save the life of patients with severe SARS-CoV-2 infection. This hypothesis, which still needs to be confirmed using appropriate preclinical models and clinical studies, gives hope for rescuing the lung vasculature functionality and reducing inflammation, respiratory distress, and mortality in severe COVID-19 patients as well as in other severe acute respiratory diseases. 

## Data Availability

Data supporting the reported results can be obtained from the corresponding author on request.

## Supplementary Materials

The following supporting information can be downloaded at: https://www.mdpi.com/article/10.3390/ijms241713088/s1. Reference [115] is cited in the supplementary file.

## References

[1] Hu B., Guo H., Zhou P., Shi Z.L. (2021). Characteristics of SARS-CoV-2 and COVID-19. Nat. Rev. Microbiol..

[2] Wang J., Jiang M., Chen X., Montaner L.J. (2020). Cytokine storm and leukocyte changes in mild versus severe SARS-CoV-2 infection: Review of 3939 COVID-19 patients in China and emerging pathogenesis and therapy concepts. J. Leukoc. Biol..

[3] Celik E., Nelles C., Kottlors J., Fervers P., Goertz L., Pinto Dos Santos D., Achenbach T., Maintz D., Persigehl T. (2022). Quantitative determination of pulmonary emphysema in follow-up LD-CTs of patients with COVID-19 infection. PLoS ONE.

[4] Chen W., Pan J.Y. (2021). Anatomical and Pathological Observation and Analysis of SARS and COVID-19: Microthrombosis Is the Main Cause of Death. Biol. Proced. Online.

[5] Merdji H., Mayeur S., Schenck M., Oulehri W., Clere-Jehl R., Cunat S., Herbrecht J.E., Janssen-Langenstein R., Nicolae A., Helms J. (2021). Histopathological features in fatal COVID-19 acute respiratory distress syndrome. Med. Intensiv. Engl. Ed..

[6] Poor H.D. (2021). Pulmonary Thrombosis and Thromboembolism in COVID-19. Chest.

[7] Channappanavar R., Perlman S. (2017). Pathogenic human coronavirus infections: Causes and consequences of cytokine storm and immunopathology. Semin. Immunopathol..

[8] Li Q., Xu W., Li W.X., Huang C.L., Chen L. (2020). Dynamics of cytokines and lymphocyte subsets associated with the poor prognosis of severe COVID-19. Eur. Rev. Med. Pharmacol. Sci..

[9] Fajgenbaum D.C., June C.H. (2020). Cytokine Storm. N. Engl. J. Med..

[10] Pons S., Arnaud M., Loiselle M., Arrii E., Azoulay E., Zafrani L. (2020). Immune Consequences of Endothelial Cells’ Activation and Dysfunction During Sepsis. Crit. Care Clin..

[11] Siddiqi H.K., Libby P., Ridker P.M. (2021). COVID-19—A vascular disease. Trends Cardiovasc. Med..

[12] To E.E., Vlahos R., Luong R., Halls M.L., Reading P.C., King P.T., Chan C., Drummond G.R., Sobey C.G., Broughton B. (2017). Endosomal NOX2 oxidase exacerbates virus pathogenicity and is a target for antiviral therapy. Nat. Commun..

[13] Taniyama Y., Griendling K.K. (2003). Reactive oxygen species in the vasculature: Molecular and cellular mechanisms. Hypertension.

[14] Reynolds A.S., Lee A.G., Renz J., DeSantis K., Liang J., Powell C.A., Ventetuolo C.E., Poor H.D. (2020). Pulmonary Vascular Dilatation Detected by Automated Transcranial Doppler in COVID-19 Pneumonia. Am. J. Respir. Crit. Care Med..

[15] Bergers G., Song S. (2005). The role of pericytes in blood-vessel formation and maintenance. Neuro Oncol..

[16] Barron L., Gharib S.A., Duffield J.S. (2016). Lung Pericytes and Resident Fibroblasts: Busy Multitaskers. Am. J. Pathol..

[17] Muhl L., He L., Sun Y., Andaloussi Mae M., Pietila R., Liu J., Genove G., Zhang L., Xie Y., Leptidis S. (2022). The SARS-CoV-2 receptor ACE2 is expressed in mouse pericytes but not endothelial cells: Implications for COVID-19 vascular research. Stem Cell Rep..

[18] He L., Mäe M.A., Sun Y., Muhl L., Nahar K., Liébanas E.V., Fagerlund M.J., Oldner A., Liu J., Genové G. (2020). Pericyte-specific vascular expression of SARS-CoV-2 receptor ACE2—Implications for microvascular inflammation and hypercoagulopathy in COVID-19. bioRxiv.

[19] Avolio E., Carrabba M., Milligan R., Kavanagh Williamson M., Beltrami A.P., Gupta K., Elvers K.T., Gamez M., Foster R.R., Gillespie K. (2021). The SARS-CoV-2 Spike protein disrupts human cardiac pericytes function through CD147 receptor-mediated signalling: A potential non-infective mechanism of COVID-19 microvascular disease. Clin. Sci..

[20] Cardot-Leccia N., Hubiche T., Dellamonica J., Burel-Vandenbos F., Passeron T. (2020). Pericyte alteration sheds light on micro-vasculopathy in COVID-19 infection. Intensive Care Med..

[21] Margo Daems L.L., Cuijpers I., Boudewijns R., Raman J., Simmonds S., Geuens N., Lox M., Verhamme P., Van Linthout S., Heymans S. (2020). SARS-CoV-2 infection leads to cardiac pericyte loss, fibrosis, cardiomyocyte hypertrophy, and diastolic dysfunction. Res. Sq..

[22] Hashimoto T., Pittet J.F. (2006). Angiopoietin-2: Modulator of vascular permeability in acute lung injury?. PLoS Med..

[23] Li F., Yin R., Guo Q. (2020). Circulating angiopoietin-2 and the risk of mortality in patients with acute respiratory distress syndrome: A systematic review and meta-analysis of 10 prospective cohort studies. Ther. Adv. Respir. Dis..

[24] van der Heijden M., van Nieuw Amerongen G.P., Koolwijk P., van Hinsbergh V.W., Groeneveld A.B. (2008). Angiopoietin-2, permeability oedema, occurrence and severity of ALI/ARDS in septic and non-septic critically ill patients. Thorax.

[25] Villa E., Critelli R., Lasagni S., Melegari A., Curatolo A., Celsa C., Romagnoli D., Melegari G., Pivetti A., Di Marco L. (2021). Dynamic angiopoietin-2 assessment predicts survival and chronic course in hospitalized patients with COVID-19. Blood Adv..

[26] Teuwen L.A., Geldhof V., Pasut A., Carmeliet P. (2020). COVID-19: The vasculature unleashed. Nat. Rev. Immunol..

[27] Pang J., Xu F., Aondio G., Li Y., Fumagalli A., Lu M., Valmadre G., Wei J., Bian Y., Canesi M. (2021). Efficacy and tolerability of bevacizumab in patients with severe COVID-19. Nat. Commun..

[28] Sahebnasagh A., Nabavi S.M., Kashani H., Abdollahian S., Habtemariam S., Rezabakhsh A. (2021). Anti-VEGF agents: As appealing targets in the setting of COVID-19 treatment in critically ill patients. Int. Immunopharmacol..

[29] Li X., Sun X., Carmeliet P. (2019). Hallmarks of Endothelial Cell Metabolism in Health and Disease. Cell Metab..

[30] Marfia G., Navone S., Guarnaccia L., Campanella R., Mondoni M., Locatelli M., Barassi A., Fontana L., Palumbo F., Garzia E. (2021). Decreased serum level of sphingosine-1-phosphate: A novel predictor of clinical severity in COVID-19. EMBO Mol. Med..

[31] Proia R.L., Hla T. (2015). Emerging biology of sphingosine-1-phosphate: Its role in pathogenesis and therapy. J. Clin. Investig..

[32] Spiegel S., Milstien S. (2003). Sphingosine-1-phosphate: An enigmatic signalling lipid. Nat. Rev. Mol. Cell Biol..

[33] Cartier A., Hla T. (2019). Sphingosine 1-phosphate: Lipid signaling in pathology and therapy. Science.

[34] Blaho V.A., Hla T. (2014). An update on the biology of sphingosine 1-phosphate receptors. J. Lipid Res..

[35] Aoki H., Aoki M., Katsuta E., Ramanathan R., Idowu M.O., Spiegel S., Takabe K. (2016). Host sphingosine kinase 1 worsens pancreatic cancer peritoneal carcinomatosis. J. Surg. Res..

[36] Kumar A., Zamora-Pineda J., Degagne E., Saba J.D. (2017). S1P Lyase Regulation of Thymic Egress and Oncogenic Inflammatory Signaling. Mediat. Inflamm..

[37] Kumar A., Saba J.D. (2015). Regulation of Immune Cell Migration by Sphingosine-1-Phosphate. Cell Mol. Biol. OMICS.

[38] Cyster J.G., Schwab S.R. (2012). Sphingosine-1-phosphate and lymphocyte egress from lymphoid organs. Annu. Rev. Immunol..

[39] Forster R., Davalos-Misslitz A.C., Rot A. (2008). CCR7 and its ligands: Balancing immunity and tolerance. Nat. Rev. Immunol..

[40] Schwab S.R., Pereira J.P., Matloubian M., Xu Y., Huang Y., Cyster J.G. (2005). Lymphocyte sequestration through S1P lyase inhibition and disruption of S1P gradients. Science.

[41] Zemann B., Kinzel B., Muller M., Reuschel R., Mechtcheriakova D., Urtz N., Bornancin F., Baumruker T., Billich A. (2006). Sphingosine kinase type 2 is essential for lymphopenia induced by the immunomodulatory drug FTY720. Blood.

[42] Ebenezer D.L., Fu P., Suryadevara V., Zhao Y., Natarajan V. (2017). Epigenetic regulation of pro-inflammatory cytokine secretion by sphingosine 1-phosphate (S1P) in acute lung injury: Role of S1P lyase. Adv. Biol. Regul..

[43] Dauphinee S.M., Karsan A. (2006). Lipopolysaccharide signaling in endothelial cells. Lab. Investig..

[44] Petrache I., Kamocki K., Poirier C., Pewzner-Jung Y., Laviad E.L., Schweitzer K.S., Van Demark M., Justice M.J., Hubbard W.C., Futerman A.H. (2013). Ceramide synthases expression and role of ceramide synthase-2 in the lung: Insight from human lung cells and mouse models. PLoS ONE.

[45] Goggel R., Winoto-Morbach S., Vielhaber G., Imai Y., Lindner K., Brade L., Brade H., Ehlers S., Slutsky A.S., Schutze S. (2004). PAF-mediated pulmonary edema: A new role for acid sphingomyelinase and ceramide. Nat. Med..

[46] Ryan A.J., McCoy D.M., McGowan S.E., Salome R.G., Mallampalli R.K. (2003). Alveolar sphingolipids generated in response to TNF-alpha modifies surfactant biophysical activity. J. Appl. Physiol..

[47] Petrache I., Natarajan V., Zhen L., Medler T.R., Richter A.T., Cho C., Hubbard W.C., Berdyshev E.V., Tuder R.M. (2005). Ceramide upregulation causes pulmonary cell apoptosis and emphysema-like disease in mice. Nat. Med..

[48] Khodadoust M.M. (2021). Inferring a causal relationship between ceramide levels and COVID-19 respiratory distress. Sci. Rep..

[49] Sammani S., Moreno-Vinasco L., Mirzapoiazova T., Singleton P.A., Chiang E.T., Evenoski C.L., Wang T., Mathew B., Husain A., Moitra J. (2010). Differential effects of sphingosine 1-phosphate receptors on airway and vascular barrier function in the murine lung. Am. J. Respir. Cell Mol. Biol..

[50] Sawai H., Hannun Y.A. (1999). Ceramide and sphingomyelinases in the regulation of stress responses. Chem. Phys. Lipids.

[51] Usui S., Sugimoto N., Takuwa N., Sakagami S., Takata S., Kaneko S., Takuwa Y. (2004). Blood lipid mediator sphingosine 1-phosphate potently stimulates platelet-derived growth factor-A and -B chain expression through S1P1-Gi-Ras-MAPK-dependent induction of Kruppel-like factor 5. J. Biol. Chem..

[52] Paik J.H., Skoura A., Chae S.S., Cowan A.E., Han D.K., Proia R.L., Hla T. (2004). Sphingosine 1-phosphate receptor regulation of N-cadherin mediates vascular stabilization. Genes. Dev..

[53] Lee M.J., Thangada S., Claffey K.P., Ancellin N., Liu C.H., Kluk M., Volpi M., Sha’afi R.I., Hla T. (1999). Vascular endothelial cell adherens junction assembly and morphogenesis induced by sphingosine-1-phosphate. Cell.

[54] Karam M., Ives A., Auclair C. (2022). Is Sphingosine-1-Phosphate a Regulator of Tumor Vascular Functionality?. Cancers.

[55] Ribatti D., Nico B., Crivellato E. (2011). The role of pericytes in angiogenesis. Int. J. Dev. Biol..

[56] McGuire P.G., Rangasamy S., Maestas J., Das A. (2011). Pericyte-derived sphingosine 1-phosphate induces the expression of adhesion proteins and modulates the retinal endothelial cell barrier. Arterioscler. Thromb. Vasc. Biol..

[57] Gerhardt H., Wolburg H., Redies C. (2000). N-cadherin mediates pericytic-endothelial interaction during brain angiogenesis in the chicken. Dev. Dyn..

[58] Li F., Wang J., Zhu Y., Liu L., Feng W., Shi W., Wang Q., Zhang Q., Chai L., Li M. (2018). SphK1/S1P Mediates PDGF-Induced Pulmonary Arterial Smooth Muscle Cell Proliferation via miR-21/BMPRII/Id1 Signaling Pathway. Cell Physiol. Biochem..

[59] Sysol J.R., Natarajan V., Machado R.F. (2016). PDGF induces SphK1 expression via Egr-1 to promote pulmonary artery smooth muscle cell proliferation. Am. J. Physiol. Cell Physiol..

[60] Waters C., Sambi B., Kong K.C., Thompson D., Pitson S.M., Pyne S., Pyne N.J. (2003). Sphingosine 1-phosphate and platelet-derived growth factor (PDGF) act via PDGF beta receptor-sphingosine 1-phosphate receptor complexes in airway smooth muscle cells. J. Biol. Chem..

[61] Gaengel K., Niaudet C., Hagikura K., Lavina B., Muhl L., Hofmann J.J., Ebarasi L., Nystrom S., Rymo S., Chen L.L. (2012). The sphingosine-1-phosphate receptor S1PR1 restricts sprouting angiogenesis by regulating the interplay between VE-cadherin and VEGFR2. Dev. Cell.

[62] Kunkel G.T., Maceyka M., Milstien S., Spiegel S. (2013). Targeting the sphingosine-1-phosphate axis in cancer, inflammation and beyond. Nat. Rev. Drug Discov..

[63] Botts S.R., Fish J.E., Howe K.L. (2021). Dysfunctional Vascular Endothelium as a Driver of Atherosclerosis: Emerging Insights into Pathogenesis and Treatment. Front. Pharmacol..

[64] Demos C., Williams D., Jo H. (2020). Disturbed Flow Induces Atherosclerosis by Annexin A2-Mediated Integrin Activation. Circ. Res..

[65] Dzobo K.E., Hanford K.M.L., Kroon J. (2021). Vascular Metabolism as Driver of Atherosclerosis: Linking Endothelial Metabolism to Inflammation. Immunometabolism.

[66] Wilhelm D.L. (1973). Mechanisms responsible for increased vascular permeability in acute inflammation. Agents Actions.

[67] Camerer E., Regard J.B., Cornelissen I., Srinivasan Y., Duong D.N., Palmer D., Pham T.H., Wong J.S., Pappu R., Coughlin S.R. (2009). Sphingosine-1-phosphate in the plasma compartment regulates basal and inflammation-induced vascular leak in mice. J. Clin. Investig..

[68] Obinata H., Hla T. (2012). Sphingosine 1-phosphate in coagulation and inflammation. Semin. Immunopathol..

[69] Oo M.L., Chang S.H., Thangada S., Wu M.T., Rezaul K., Blaho V., Hwang S.I., Han D.K., Hla T. (2011). Engagement of S1P(1)-degradative mechanisms leads to vascular leak in mice. J. Clin. Investig..

[70] Garcia J.G., Liu F., Verin A.D., Birukova A., Dechert M.A., Gerthoffer W.T., Bamberg J.R., English D. (2001). Sphingosine 1-phosphate promotes endothelial cell barrier integrity by Edg-dependent cytoskeletal rearrangement. J. Clin. Investig..

[71] Bogatcheva N.V., Verin A.D. (2008). The role of cytoskeleton in the regulation of vascular endothelial barrier function. Microvasc. Res..

[72] Ruiz M., Frej C., Holmer A., Guo L.J., Tran S., Dahlback B. (2017). High-Density Lipoprotein-Associated Apolipoprotein M Limits Endothelial Inflammation by Delivering Sphingosine-1-Phosphate to the Sphingosine-1-Phosphate Receptor 1. Arterioscler. Thromb. Vasc. Biol..

[73] Frej C., Linder A., Happonen K.E., Taylor F.B., Lupu F., Dahlback B. (2016). Sphingosine 1-phosphate and its carrier apolipoprotein M in human sepsis and in Escherichia coli sepsis in baboons. J. Cell. Mol. Med..

[74] Juhasz L., Lorincz H., Szentpeteri A., Nadro B., Varga E., Paragh G., Harangi M. (2022). Sphingosine 1-Phosphate and Apolipoprotein M Levels and Their Correlations with Inflammatory Biomarkers in Patients with Untreated Familial Hypercholesterolemia. Int. J. Mol. Sci..

[75] Christoffersen C., Obinata H., Kumaraswamy S.B., Galvani S., Ahnstrom J., Sevvana M., Egerer-Sieber C., Muller Y.A., Hla T., Nielsen L.B. (2011). Endothelium-protective sphingosine-1-phosphate provided by HDL-associated apolipoprotein M. Proc. Natl. Acad. Sci. USA.

[76] Poti F., Simoni M., Nofer J.R. (2014). Atheroprotective role of high-density lipoprotein (HDL)-associated sphingosine-1-phosphate (S1P). Cardiovasc. Res..

[77] Therond P., Chapman M.J. (2022). Sphingosine-1-phosphate: Metabolism, transport, atheroprotection and effect of statin treatment. Curr. Opin. Lipidol..

[78] Winkler M.S., Claus R.A., Schilder M., Pohlmann S., Coldewey S.M., Grundmann J., Fricke T., Moerer O., Meissner K., Bauer M. (2021). Erythrocytes increase endogenous sphingosine 1-phosphate levels as an adaptive response to SARS-CoV-2 infection. Clin. Sci..

[79] Winkler M.S., Martz K.B., Nierhaus A., Daum G., Schwedhelm E., Kluge S., Graler M.H. (2019). Loss of sphingosine 1-phosphate (S1P) in septic shock is predominantly caused by decreased levels of high-density lipoproteins (HDL). J. Intensive Care.

[80] Wilkerson B.A., Grass G.D., Wing S.B., Argraves W.S., Argraves K.M. (2012). Sphingosine 1-phosphate (S1P) carrier-dependent regulation of endothelial barrier: High density lipoprotein (HDL)-S1P prolongs endothelial barrier enhancement as compared with albumin-S1P via effects on levels, trafficking, and signaling of S1P1. J. Biol. Chem..

[81] Chun J., Giovannoni G., Hunter S.F. (2021). Sphingosine 1-phosphate Receptor Modulator Therapy for Multiple Sclerosis: Differential Downstream Receptor Signalling and Clinical Profile Effects. Drugs.

[82] Cohan S., Lucassen E., Smoot K., Brink J., Chen C. (2020). Sphingosine-1-Phosphate: Its Pharmacological Regulation and the Treatment of Multiple Sclerosis: A Review Article. Biomedicines.

[83] McGinley M.P., Cohen J.A. (2021). Sphingosine 1-phosphate receptor modulators in multiple sclerosis and other conditions. Lancet.

[84] Roy R., Alotaibi A.A., Freedman M.S. (2021). Sphingosine 1-Phosphate Receptor Modulators for Multiple Sclerosis. CNS Drugs.

[85] Tsai H.C., Han M.H. (2016). Sphingosine-1-Phosphate (S1P) and S1P Signaling Pathway: Therapeutic Targets in Autoimmunity and Inflammation. Drugs.

[86] Chun J., Hartung H.P. (2010). Mechanism of action of oral fingolimod (FTY720) in multiple sclerosis. Clin. Neuropharmacol..

[87] Chun J., Kihara Y., Jonnalagadda D., Blaho V.A. (2019). Fingolimod: Lessons Learned and New Opportunities for Treating Multiple Sclerosis and Other Disorders. Annu. Rev. Pharmacol. Toxicol..

[88] Hach T., Shakeri-Nejad K., Bigaud M., Dahlke F., de Micco M., Petricoul O., Graham G., Piani-Meier D., Turrini R., Brinkmann V. (2023). Rationale for Use of Sphingosine-1-Phosphate Receptor Modulators in COVID-19 Patients: Overview of Scientific Evidence. J. Interferon Cytokine Res..

[89] Tasat D.R., Yakisich J.S. (2021). Rationale for the use of sphingosine analogues in COVID-19 patients. Clin. Med..

[90] Vahed S.Z., Ghiyasvand S., Khatibi S.M., Patel B., Shoja M.M., Tolouian R., Ardalan M. (2021). Sphingosine 1 phosphate agonists (SPI); a potential agent to prevent acute lung injury in COVID-19. Immunopathol. Persa.

[91] Muller H.C., Hocke A.C., Hellwig K., Gutbier B., Peters H., Schonrock S.M., Tschernig T., Schmiedl A., Hippenstiel S., N’Guessan P.D. (2011). The Sphingosine-1 Phosphate receptor agonist FTY720 dose dependently affected endothelial integrity in vitro and aggravated ventilator-induced lung injury in mice. Pulm. Pharmacol. Ther..

[92] Natarajan V., Dudek S.M., Jacobson J.R., Moreno-Vinasco L., Huang L.S., Abassi T., Mathew B., Zhao Y., Wang L., Bittman R. (2013). Sphingosine-1-phosphate, FTY720, and sphingosine-1-phosphate receptors in the pathobiology of acute lung injury. Am. J. Respir. Cell Mol. Biol..

[93] Shea B.S., Brooks S.F., Fontaine B.A., Chun J., Luster A.D., Tager A.M. (2010). Prolonged exposure to sphingosine 1-phosphate receptor-1 agonists exacerbates vascular leak, fibrosis, and mortality after lung injury. Am. J. Respir. Cell Mol. Biol..

[94] Teymouri S., Pourbayram Kaleybar S., Hejazian S.S., Hejazian S.M., Ansarin K., Ardalan M., Zununi Vahed S. (2023). The effect of Fingolimod on patients with moderate to severe COVID-19. Pharmacol. Res. Perspect..

[95] Liu S., Ni C., Zhang D., Sun H., Dong X., Che N., Liang X., Chen C., Liu F., Bai J. (2019). S1PR1 regulates the switch of two angiogenic modes by VE-cadherin phosphorylation in breast cancer. Cell Death Dis..

[96] Bandhuvula P., Saba J.D. (2007). Sphingosine-1-phosphate lyase in immunity and cancer: Silencing the siren. Trends Mol. Med..

[97] Ohtoyo M., Tamura M., Machinaga N., Muro F., Hashimoto R. (2015). Sphingosine 1-phosphate lyase inhibition by 2-acetyl-4-(tetrahydroxybutyl)imidazole (THI) under conditions of vitamin B6 deficiency. Mol. Cell. Biochem..

[98] Bagdanoff J.T., Donoviel M.S., Nouraldeen A., Carlsen M., Jessop T.C., Tarver J., Aleem S., Dong L., Zhang H., Boteju L. (2010). Inhibition of sphingosine 1-phosphate lyase for the treatment of rheumatoid arthritis: Discovery of (E)-1-(4-((1R,2S,3R)-1,2,3,4-tetrahydroxybutyl)-1H-imidazol-2-yl)ethanone oxime (LX2931) and (1R,2S,3R)-1-(2-(isoxazol-3-yl)-1H-imidazol-4-yl)butane-1,2,3,4-tetraol (LX2932). J. Med. Chem..

[99] Longo V., Brunetti O., Gnoni A., Cascinu S., Gasparini G., Lorusso V., Ribatti D., Silvestris N. (2016). Angiogenesis in pancreatic ductal adenocarcinoma: A controversial issue. Oncotarget.

[100] Tabasinezhad M., Samadi N., Ghanbari P., Mohseni M., Saei A.A., Sharifi S., Saeedi N., Pourhassan A. (2013). Sphingosin 1-phosphate contributes in tumor progression. J. Cancer Res. Ther..

[101] Cheung Y.P., Mastrullo V., Maselli D., Butsabong T., Madeddu P., Maringer K., Campagnolo P. (2020). A Critical Role for Perivascular Cells in Amplifying Vascular Leakage Induced by Dengue Virus Nonstructural Protein 1. mSphere.

[102] Caldera-Crespo L.A., Paidas M.J., Roy S., Schulman C.I., Kenyon N.S., Daunert S., Jayakumar A.R. (2021). Experimental Models of COVID-19. Front. Cell Infect. Microbiol..

[103] Chu H., Chan J.F., Yuen K.Y. (2022). Animal models in SARS-CoV-2 research. Nat. Methods.

[104] Zheng J., Wong L.R., Li K., Verma A.K., Ortiz M.E., Wohlford-Lenane C., Leidinger M.R., Knudson C.M., Meyerholz D.K., McCray P.B. (2021). COVID-19 treatments and pathogenesis including anosmia in K18-hACE2 mice. Nature.

[105] Hassan A.O., Case J.B., Winkler E.S., Thackray L.B., Kafai N.M., Bailey A.L., McCune B.T., Fox J.M., Chen R.E., Alsoussi W.B. (2020). A SARS-CoV-2 Infection Model in Mice Demonstrates Protection by Neutralizing Antibodies. Cell.

[106] Sia S.F., Yan L.M., Chin A.W.H., Fung K., Choy K.T., Wong A.Y.L., Kaewpreedee P., Perera R., Poon L.L.M., Nicholls J.M. (2020). Pathogenesis and transmission of SARS-CoV-2 in golden hamsters. Nature.

[107] Rockx B., Kuiken T., Herfst S., Bestebroer T., Lamers M.M., Oude Munnink B.B., de Meulder D., van Amerongen G., van den Brand J., Okba N.M.A. (2020). Comparative pathogenesis of COVID-19, MERS, and SARS in a nonhuman primate model. Science.

[108] Salguero F.J., White A.D., Slack G.S., Fotheringham S.A., Bewley K.R., Gooch K.E., Longet S., Humphries H.E., Watson R.J., Hunter L. (2021). Comparison of rhesus and cynomolgus macaques as an infection model for COVID-19. Nat. Commun..

[109] Munster V.J., Feldmann F., Williamson B.N., van Doremalen N., Perez-Perez L., Schulz J., Meade-White K., Okumura A., Callison J., Brumbaugh B. (2020). Respiratory disease in rhesus macaques inoculated with SARS-CoV-2. Nature.

[110] Woolsey C., Borisevich V., Prasad A.N., Agans K.N., Deer D.J., Dobias N.S., Heymann J.C., Foster S.L., Levine C.B., Medina L. (2020). Establishment of an African green monkey model for COVID-19. bioRxiv.

[111] Singh D.K., Singh B., Ganatra S.R., Gazi M., Cole J., Thippeshappa R., Alfson K.J., Clemmons E., Gonzalez O., Escobedo R. (2021). Responses to acute infection with SARS-CoV-2 in the lungs of rhesus macaques, baboons and marmosets. Nat. Microbiol..

[112] Bao L., Deng W., Huang B., Gao H., Liu J., Ren L., Wei Q., Yu P., Xu Y., Qi F. (2020). The pathogenicity of SARS-CoV-2 in hACE2 transgenic mice. Nature.

[113] Zhang Y., Huang K., Wang T., Deng F., Gong W., Hui X., Zhao Y., He X., Li C., Zhang Q. (2021). SARS-CoV-2 rapidly adapts in aged BALB/c mice and induces typical pneumonia. J. Virol..

[114] Kim Y.I., Kim S.G., Kim S.M., Kim E.H., Park S.J., Yu K.M., Chang J.H., Kim E.J., Lee S., Casel M.A.B. (2020). Infection and Rapid Transmission of SARS-CoV-2 in Ferrets. Cell Host Microbe.

[115] Ackermann M., Carvajal I.M., Morse B.A., Moreta M., O’Neil S., Kossodo S., Peterson J.D., Delventhal V., Marsh H.N., Furfine E.S. (2011). Adnectin CT-322 inhibits tumor growth and affects microvascular architecture and function in Colo205 tumor xenografts. Int. J. Oncol..

