# Supplementary material for: Sphingosine-1-Phosphate as Lung and Cardiac Vasculature Protecting Agent in SARS-CoV-2 Infection
Source: Int J Mol Sci. 2023 Aug 23;24(17):13088. doi: 10.3390/ijms241713088 (PMC10488186; doi:10.3390/ijms241713088)
Supplement: Supplementary file 1 [file ijms-24-13088-s001.zip › ijms-2495679-supplementary.pdf]

Supplementary File 1

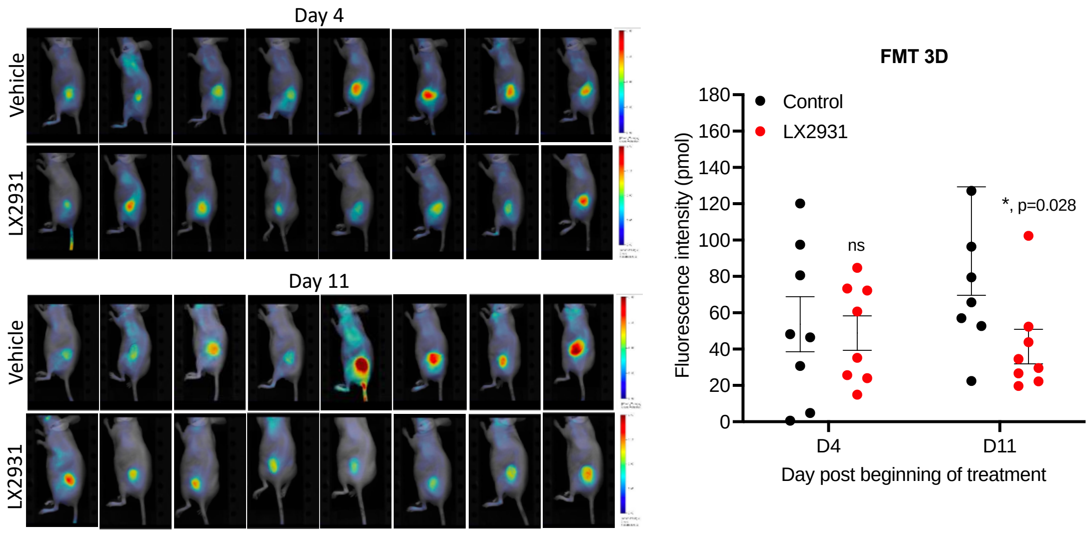

The S1P lyase inhibitor LX2931 significantly reduces blood vessel permeability and leakage in experimental pancreatic tumor

Human pancreatic MiaPaCa2 cancer cells were subcutaneous implanted into the left flank of nude mice. When tumors reached approximately 100-300 mm^3, animals were randomized in two groups (n=8 per group) and the treatment started (D0). Animals were treated 4 days per week (D0-D3 and D7-D10) for 2 weeks with vehicle or LX2931 (30 mg/kg). At D3 and D10 animals were injected (IV) by fluorescent vascular probe AngioSense 750 EX and then anesthetized and imaged 24h after injection by Fluorescence Molecular Imaging (FMT) at D4 and D11. The collected fluorescence data were reconstructed by FMT system software as 3D fluorescence datasets showing signal within the flank tumors. To quantify this tumor fluorescence, 3D regions of interest (ROI) were drawn encompassing the tumors. The fluorescence intensity indicates the accumulation of the AngioSense 750 EX molecule in areas of vascular leakage (Ackermann et al 2011 *Int J Oncol*). Images of vehicle vs. LX2931 treated mice are shown. The graph represents the individual and mean fluorescence intensity ± SEM. P values were calculated using Mann-Whitney test. For more details, see "Materials and Methods" below.

Materials and Methods

Animals

Female athymic nude mice (BALB/cAnNRj-Foxn1^{nu}/Foxn1^{nu}), 6- to 7-week-old on the first day of the experimental phase, were obtained from Janvier Labs (Le Genest St Isle, France).
Animals were maintained in specific pathogen-free animal housing at the SFP animal facility: Zone d'Evaluation Fonctionnelle (ZEF), Canal Biotech 2, 3 rue des Satellites, Parc Technologique du Canal, 31400 Toulouse, France, under the responsibility of Imavita. Animals were delivered 7 days before the experiment for acclimatizing to environmental conditions. Animals were individually identified using a number written/tattooed on the tail and by tags on cages. Animal diet consisted of AIN93G Nude -B6 pellets (SAFE SAS, Augy, France). Mice were offered sterilized tap water ad libitum throughout the study.

The *in vivo* design and procedures were assessed and authorized by Ethical Committee N°CEEA-122 (Imavita ethical project identification N°IMV-ETH-12). The animal care, housing and experiments comply with the recommendations of Directive 86/609/EEC. Animal facilities have agreement obtained from the French Veterinary Authorities.

Cell culture

Human pancreatic MiaPaCa2 cancer cells (property Imavita/Origin ECACC (85062806)) were cultured in Dulbecco modified Eagle medium supplemented with 10% fetal bovine serum and 2 mM L-glutamine and maintained in a humidified atmosphere of 5 % CO_2 in air at 37°C.

Tumor induction

Tumor induction was performed by implanting $2x10^6$ MiaPaCa2 cells in 0.1 mL of PBS:Matrigel (1:1) by subcutaneous injection in the left flank of female Balb/c nude mice.
Tumor implantation was done under isoflurane anesthesia and aseptic conditions.
Tumor engraftment was monitored by daily observation and palpation. Caliper measurement was performed three times a week since tumors were palpable. Tumor size in mm^3 was estimated from the formula $w^2 \times l \times \pi/6$ where "*l*" is the longest diameter of the tumor and "*w*" is the diameter perpendicular to the longest diameter measured in millimeters.

Test compound formulation

The test item LX2931 (batch #ACB-UCB-0419) compound was manufactured at the department of chemistry at University British Columbia (Canada) and stored at 4°C, light protected.
For treatment doses of 30 mg/kg, dosing solutions were freshly prepared by solubilizing LX2931 in deionized sterile water at 3 mg/mL. Preparations were stored at room temperature, in a light protected cabinet and used within 3 hours after preparation.

Animal allocation, treatment and assessment

When tumors reached a volume of about 100-400 mm^3, 16 mice were selected and randomly assigned to the control or LX2931 treatment groups (8 animals/group) with equivalent average starting tumor volumes. Treatment started on day 0 (D0). Treatment consisted of oral administration of LX2931 (30 mg/kg) every day for four days followed by three days without treatment. This treatment schedule was repeated for 2 weeks. Vehicle control, consisting of the diluent of LX2931; i.e. deionized sterile water, was administered to the control mice at the same volume and schedule as LX2931 compounds.
Mice were monitored for body weight, clinical signs and tumor volume three times per week.
Tumor vasculature was monitored by fluorescence tomography *in vivo* imaging.
None of the mice has reached the ethical endpoint criteria (general alteration of behavior or clinical signs; tumor volume ≥2000 mm^3, body weight loss (BWL) of ≥20%) during the experimental period. Mice were sacrificed at the end of the treatment period after last imaging time point (day 11; D11).

Fluorescence tomography *in vivo* imaging

Mice were imaged using the FMT 2500™ fluorescence tomography *in vivo* imaging system (PerkinElmer, Waltham, MA).
The fluorescent probe AngioSense 750 EX was injected intravenously in the tail vein of mice at days 3 and 10. One imaging session was performed 24h after injection of the fluorescent probe (D4 and D11). The AngioSense 750 EX Fluorescent Imaging probe (PerkinElmer) was used according to the supplier's recommendations.

For each animal 1 scan in decubitus dorsal/ventral position was performed (tumor area) with the FMT (NIR excitation laser 745 nm / emission 770-800 nm). The collected fluorescence data were reconstructed by FMT 2500 system software (TrueQuant V2.0, PerkinElmer, Waltham, MA) for the quantification of the three-dimensional fluorescence signal in whole animal body.

Three-dimensional regions of interest (ROI) were drawn encompassing the relevant biology on the tumor area. A tumor zone of acquisition was performed (total of 32 scans).

Quantitative imaging was performed on each mouse to evaluate fluorescence of Region of Interest (ROI: tumor zone). The quantity of fluorophore (AngioSense 750 EX) in ROI was determined for each mouse.

AngioSense 750 EX is constitutively fluorescent and has a MW of 250 kDa that allows monitoring of vascular leakage. It has a half-life in plasma of 6 h, is exclusively intravascular 1 h after injection and extravasates at sites of increased vascular permeability by 24 h, allowing the detection of blood pooling in tumors. Thus, the results (fluorescence intensity) indicate accumulation of the AngioSense molecule in areas of vascular leakage (Ackermann et al 2011 *Int J Oncol*).

Statistical significance of differences between experimental groups was determined by Mann-Whitney test using the GraphPad Prism 7 software (San Diego, CA, USA). Differences were considered significant at confidence levels greater than 95% ($P < 0.05$).

Reference

Ackermann, M.; Carvajal, I.M.; Morse, B.A.; Moreta, M.; O'Neil, S.; Kossodo, S.; Peterson, J.D.; Delventhal, V.; Marsh, H.N.; Furfine, E.S.; et al. Adnectin CT-322 inhibits tumor growth and affects microvascular architecture and function in Colo205 tumor xenografts. *Int J Oncol* **2011**, *38*, 71-80.
